# A Combined Offline and Online Algorithm for Real-Time and Long-Term Classification of Sheep Behaviour: Novel Approach for Precision Livestock Farming

**DOI:** 10.3390/s19143201

**Published:** 2019-07-20

**Authors:** Jorge A. Vázquez-Diosdado, Veronica Paul, Keith A Ellis, David Coates, Radhika Loomba, Jasmeet Kaler

**Affiliations:** 1 School of Veterinary Medicine and Science, University of Nottingham, Sutton Bonington LE12 5RD, UK; 2 Internet of Things Systems Research, Intel Labs, W23 CX68 Leixlip, Ireland

**Keywords:** algorithm, concept drift, Precision Livestock Farming, machine learning, animal behaviour

## Abstract

Real-time and long-term behavioural monitoring systems in precision livestock farming have huge potential to improve welfare and productivity for the better health of farm animals. However, some of the biggest challenges for long-term monitoring systems relate to “concept drift”, which occurs when systems are presented with challenging new or changing conditions, and/or in scenarios where training data is not accurately reflective of live sensed data. This study presents a combined offline algorithm and online learning algorithm which deals with concept drift and is deemed by the authors as a useful mechanism for long-term in-the-field monitoring systems. The proposed algorithm classifies three relevant sheep behaviours using information from an embedded edge device that includes tri-axial accelerometer and tri-axial gyroscope sensors. The proposed approach is for the first time reported in precision livestock behavior monitoring and demonstrates improvement in classifying relevant behaviour in sheep, in real-time, under dynamically changing conditions.

## 1. Introduction

Behavioural classification systems based on sensor technology, such as accelerometers, gyroscopes and magnetometers, have allowed researchers to obtain a deeper understanding of the behaviour, movements and social interactions of wild and domestic animals [1,2,3,4]. More specifically, in livestock agriculture production [4], sensor technology can have a positive impact for farm decision making by providing useful real-time behavioural and health information. An automated behavioural monitoring system can be used to improve the health and welfare of animals by detecting early changes in behaviour that can be linked to health changes. For example, Mathews et al. [5] were able to detect behavioural changes in pigs using an automated tracking system with depth video cameras, Pastell et al. [6] found differences in asymmetry variance and forward acceleration between lame and non-lame cows using a 3D accelerometer-based sensor, and Chapinal et al [7] were able to identify early signs of lameness using activity levels obtained with 3D accelerometers.

Despite recent advances made in several disease detection systems, there are still practical and technical challenges in delivering a complete real-time and long-term monitoring system for farm animals. Among some of the technological challenges are computational power, energy consumption and data transmission, which impact on the deployable life of a solution. Possible approaches to tackle energy consumption and computational power considerations include optimal algorithm selection, sampling size, window size, and sensor position among others [8]. Nevertheless, utilising embedded computation to classify “on-device” is a far more frugal strategy than incurring the energy penalty of data transmission which is an order of magnitude greater than computation. 

Aside from energy considerations, one of the biggest remaining technical challenges for long-term deployed behavioural monitoring is “concept drift” [9], which occurs mainly in dynamically changing conditions [10]. More specifically, concept drift occurs when a system is required to adapt to a change in data distributions within the concept. In classical supervised classification problems, it is typically assumed that the data in the design model is randomly selected from the same distribution as the points that will be classified in the future. This is quite unrealistic, and as Hoadley et al. [11] remarked “high performance on test sample does not guarantee high performance on futures samples, things do change”.

The intrinsic dynamic nature of many different classification problems can have an impact on the performance of future testing. Concept drift is a well-known phenomenon in industries such as security, police (i.e., fraud detection, insider trading detection, etc.) finance (i.e., bankruptcy prediction, etc.) among others [12]. However, in human activity recognition (HAR) researchers have only recently started to consider this phenomenon when developing classification systems [13,14,15]. For example, Abdallah et al. [13] developed a HAR algorithm that considers evolving data streams to classify sitting, lying, walking, and standing with high levels of accuracy (93.1%, 99.2%, 70.4% and 61.3%, respectively). Similarly, Tennant et al. [15], developed a Micro-Cluster Nearest Neighbour (MC-NN) classifier for data streams of activities such as biking, sitting, standing, stairs-up, stairs-down and walking, with an overall accuracy up to 94.03%. One clear example of a non-stationary learning problem is when a system is trained in one environment and tested in a different one (i.e., laboratory conditions versus real-life conditions). For example, Awais et al. [16] highlighted the gap in the performance of physical activity classification systems between laboratory and real-life scenarios. Similarly, Riou et al. [17] showed a decrement on the performance of a HAR classification algorithm when applied activities were performed in confined and unrestricted environments. Behavioural classification in animals can also show discrepancies in performance given environment variance. Such discrepancies can be due to differences in the animals (age, breed, etc.) and environmental characteristics (terrain elevation, type of soil, particular farm constrains, etc.). Discrepancies in behaviour classification performance using pedometers, much like IceTag devices, have been reported [18] with relatively high (35%) mismatch values between the proportion of steps. Guo et al. reported differences in classification performance of grazing behaviour of sheep on pasture with different sward heights [19]. Recently, Rahman et al. [20] and Smith et al. [21] showed that the lack of feature correspondence between sensors/trials can lead to poor classification accuracies in animal behaviour. These examples highlight the importance of carefully considering output measures of behavioural classifier systems under different conditions when using them to infer additional behavioural information (i.e., activity budgets, bout durations, etc.). A possible solution to deal with this type of phenomenon is to use methods that incorporate concept drift in the architecture. To the authors’ knowledge, concept drift has not been considered when designing a behavioural monitoring system using animal-borne technology. 

Available solutions in the literature that deal with concept drift can be put into two architectural groups [22]: one that uses a model that continuously adapts to changing conditions but at the risk of forgetting the already learned concepts (catastrophic forgetting effect), and another architecture that incorporates the retention of previous knowledge and newly learned concepts. Flexible architectural systems based on the latter have been proposed to efficiently deal with concept drift [23,24] including hybrid systems that combine offline and online learning algorithms [22] In these hybrid systems, the offline algorithm is completely static and it is pre-trained with offline information, while the online algorithm continues learning through the deployment lifetime utilising online information. Hence, in a hybrid offline and online system, the online algorithm deals with data distribution changes while the classifier, trained offline, provides a classification based on already learned knowledge. However, behaviour in animals is naturally a non-stationary learning problem were the distribution of the classes might differ over time. Within such systems, an optimal combination of individual classifiers can help improve performance [25] and can be performed using several different methods, such as heuristics, iterative optimization, and recently developed game theoretic procedures [26], or by meta decision trees [27]. 

In this study, a novel algorithmic solution is proposed for precision livestock farming focusing on an animal behaviour classification problem that deals with the challenge of concept drift. This algorithm is based on a combined offline and online algorithm, and the authors demonstrate that the accuracy of the approach outperforms both the offline classifier and online algorithm. The authors posit that this approach has considerable potential for improving classification accuracy in real-time deployment scenarios and therefore has considerable potential for improving animal welfare and livestock production.

The sections that follow firstly outline the algorithmic methodology, followed by details of the experimentation setup and operation and, finally, present and discuss the study results.

## 2. Algorithmic Methodology

As stated, the goal of this study was to investigate a mechanism for behavioural classification in sheep, capable of adapting to changing conditions, i.e., “concept drift”. The approach taken essentially combines offline and online algorithms. It was designed to address concept drift and to deliver better performance than a standalone offline or online approach. 

Abstractly, the online/offline algorithms act on input information from a custom-made wearable device based on the Intel® Quark™ SE C1000 microcontroller (Intel Labs, Leixlip, Ireland). The device processes tri-axial accelerometer and gyroscope data from an on-board inertial measurement unit (IMU) and outputs classifications using the integrated Pattern Matching Engine (PME).

In the proposed approach, IMU sensor data is essentially processed by two algorithms for a given time window, with a third algorithm applied to the classification labels of each. The three algorithms are presented below and high-level descriptions are provided in the following sub-sections:

1. *Offline KNN algorithm*: a vectorised K-nearest neighbours (KNN) model (trained offline) that outputs a label, i.e., walking, standing, lying. Implemented online using the PME of the SE C1000.
2. *Online k-means algorithm*: an online unsupervised vector-means clustering algorithm that produces a classification, i.e., walking, standing, lying, using the following function as an input:
  ⚬ *Online MeanAMag calculation*: a mean of acceleration magnitude (MeanAMag) for the time window, essentially can be used as an online local activity indication.
3. *Online combined algorithm:* a combined algorithm that is based on decision rules from prior historical data and applied to the output of 1 and 2 to produce an overall classification label. This is the proposed combined offline and online algorithm.

### 2.1. Offline KNN Algorithm

**Figure 1:**
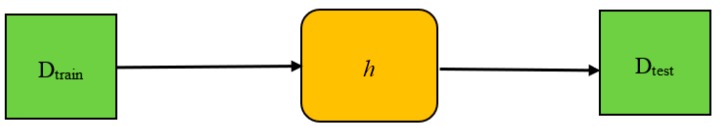
Offline learning scheme. Both train and test sets are disjoint.

In an offline learning process, an algorithm generates a model based on a training set D_train_ and afterwards the model is tested using another dataset D_test_. In this study, a feature vector model was developed in an offline process utilising previously collected data from Walton et al. [8]. The model is a representative vectorised subset of the 8455 (7 s window at 16 Hz sampling frequency) sample dataset. The Pattern Matching Engine (PME) of the SC1000 was then loaded/flashed with this model. The PME is effectively an associative memory. This associative memory is basically a bi-dimensional array (one byte per element) which contains the feature vectors coming from the offline training step. A new vector is compared to the array in parallel. In its simplest form, the closest vector in the training set is identified and the associated category retrieved. One can store up to 128 feature vectors and depending on the application one can store more than one training set at once (called “context”). The recognition rate is up in the 20 thousand recognitions per range and, by using this in-hardware mechanism, one can make very rapid and efficient classifications suited to in-the-field scenarios. Figure 1 shows a typical simplistic scheme for the offline algorithm.

### 2.2. Online K-means Algorithm 

**Figure 2:**
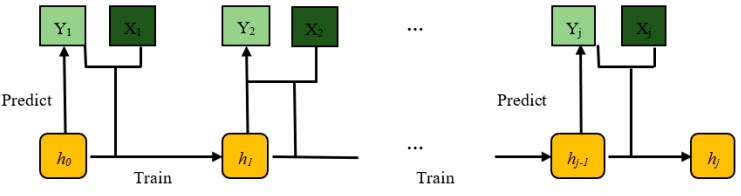
Online learning scheme.

In contrast to offline learning approaches, online learning algorithms process a potentially infinite sequence of predictors (*x_i_*) and outputs (*y_i_*) as they arrive one after the other [28]. The learning objective is to predict the current label *y_t_* for a given input *x_t_*, using the previously learned model *h_t−1_*, hence the prediction $\left(\hat{y_{t}}\right)$ is computed as $\hat{y_{t}}=h_{t-1}\left(x_{t-1}\right).$ Another difference with offline learning is that training and testing datasets are not completely disjointed, rather each instance sample is used for model testing and in the next step for model training. In Figure 2, we show a scheme of a typical online algorithm. Each time the predictor function *h* is constructed on past received information and it only predicts the following label $\hat{y_{t}}=h_{t-1}\left(x_{t-1}\right)$. 

Several different methods exist for such online learning, including incremental support vector machines, online random forest, incremental vector quantization and stochastic gradient descent, among others. In this study, we used an unsupervised k-means online clustering algorithm [29]. The unsupervised k-means online clustering algorithm was implemented in Matlab [30] using the k-means built in function. The unsupervised k-means online clustering follows the next steps:

1. Initialise the k number of centroids C_1_, C_2_, C_3_ from values obtained either from previously collected data or from an initial subset (*n* = 100 points in our case) of the current dataset.
2. Predict the behavioural class of the new MeanAMag point using the initial values for the centroids. The predicted behavioural class is obtained by finding the class with the closest (according to Euclidean distance) centroid to the MeanAMag point.
3. Update the centroids by adding the most recently acquire MeanAMag datapoint.
4. Steps 2 and 3 are repeated until no more information is provided. At each new iteration the classification prediction (Y_j_) will be obtained using the centroids from the previous iteration (C_1j−1_, C_2j−1_, C_3j−1_).

**Figure 3:**
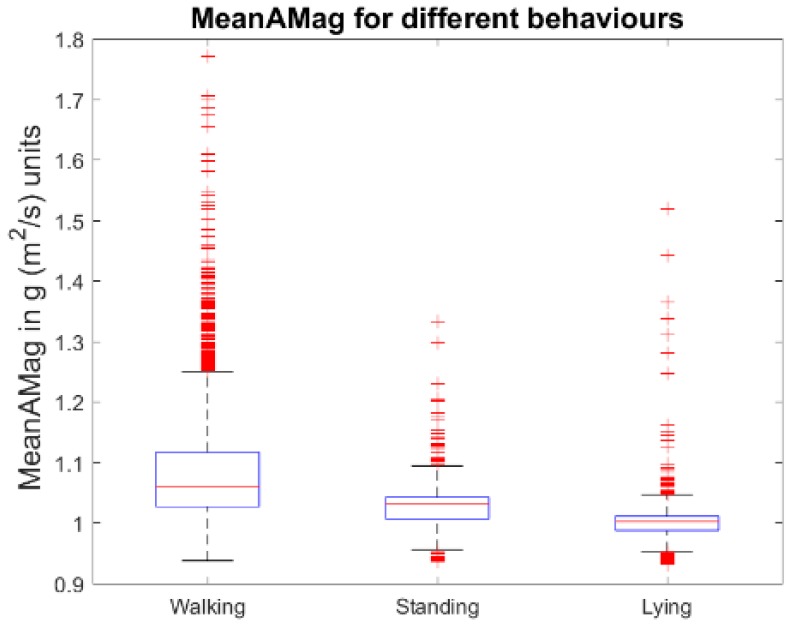
Boxplot of the MeanAMag across the different behaviours.

Within this algorithm, an online calculation for the mean of acceleration magnitude, termed MeanAMag*,* was used as an input. This variable was selected as the device supports an online local variable computation in real-time on the SE C1000 device. The following formula was used:

$$MeanAMag=\;\frac{\sum_{i=1}^{n}A_{l}}{n}$$

where $A_{l}$ represents the magnitude of the acceleration at every single sample point. The magnitude of the acceleration was defined as:

$$A_{l}=\;\sqrt{A_{x_{i}}^{2}+A_{y_{i}}^{2}+A_{z_{i}}^{2}}$$

where $A_{x_{i}}^{2},\;A_{y_{i}}^{2},\;A_{x_{i}}^{2}$ represents the acceleration for each sample point at the axes *x*, *y*, and *z*, respectively. To obtain the discretised version of the MeanAMag, the value of gravity was subtracted from the $A_{l}$ and then the values were discretised in a range between 1 and 20. Sample frequency for the accelerometer and gyroscope were set to 16 Hz. The discretised version of the MeanAMag represent a discretised version of the well-known “dynamic body acceleration”, which has been used effectively as a proxy of energy expenditure in animals [31] and as a one of the only two feature characteristics required to classify different behaviours in cows [32]. Additionally, the discretised MeanAMag calculation supports low computational complexity and demonstrates observable differences across the different behaviours from the previously collected data [8], as shown in Figure 3.

The classification power of this measure can be observed in Figure 2 in Walton et al. [8]. Window size of the classification output from the device was set to 7 s with a 3 s delay between readings, hence producing a timestamp, predicted behaviour label (PME classification) and a MeanAMag output every 10 s.

### 2.3. Combined Offline and Online Algorithm

**Figure 4:**
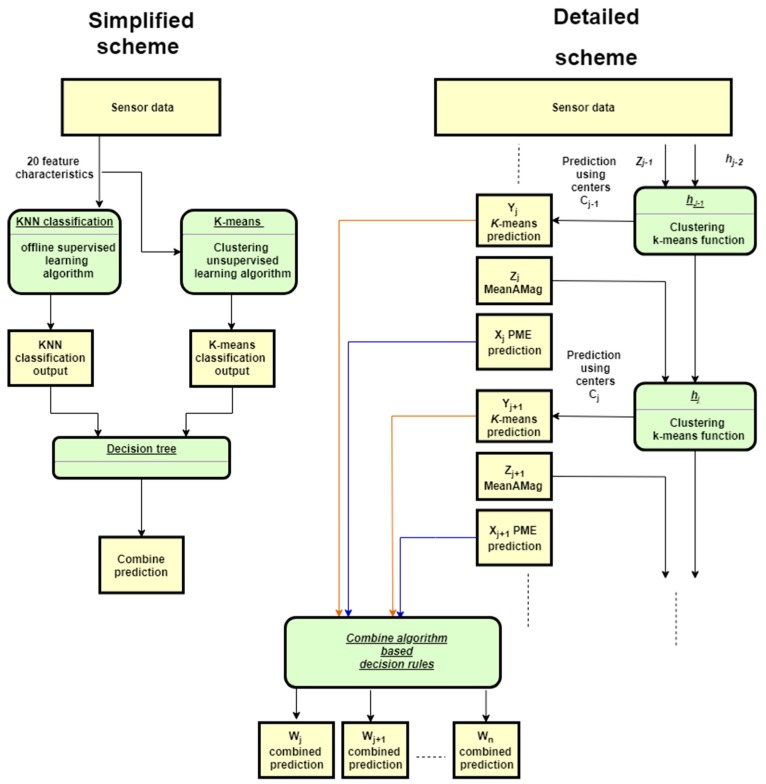
Combined offline and online learning scheme. In this scheme KNN refers to the K-nearest neighbours model (trained offline).

As stated, this study proposes an approach that combines the classification labels from the offline KNN algorithm (implemented via the PME) and the online *K*-means algorithm, which uses the online MeanAMag calculation as an input, with both acting as inputs to a set of decision rules. An implementation scheme of the overall algorithmic approach is shown in Figure 4 and described in the following steps:

1. Simplified “in operation” workflow:
  1.1 Raw sensor data is captured and variables calculated.
  1.2 Feature characteristics are calculated, including MeanAMag, and a vector to classify is created.
  1.3 Vector to classify is fed to the KNN supervised classification algorithm.
  1.4 MeanAMag is sent to the K-means unsupervised learning algorithm.
  1.5 Outputs of the KNN and K-means algorithms are used in a decision tree algorithm for the classification of the three different behaviours.
2. Detailed “in operation” workflow:
  2.1 The dataset generated for this study (in Figure 4) was based on acceleration and gyroscope data sampled at 16 Hz over a 7 s window size.
  2.2 At every single window (i), 4 variables were calculated, namely, acceleration magnitude, acceleration magnitude difference, gyroscope magnitude and gyroscope magnitude difference.
  2.3 Five feature characteristics, namely, mean, standard deviation, interquartile range, kurtosis and min, are applied to the four variables; this gives 20 feature characteristics used to create a vector to be classified (one of which is MeanAMag).
  2.4 The vector to be classified is sent to the PME which encompasses the offline implemented KNN vector model and a *PME_Classifcation_label* is returned (X_i_).
  2.5 The MeanAMag feature characteristic *Z*_i_ (calculated at step 2.3)_,_ is used to predict a class label from the k-means $(e.g.,\;y_{j}=h_{j-1}\left(z_{j-1}\right)=\;Y_{j}$). The predicted behavioural class is obtained by finding the class with the closest (according to Euclidean distance) centroid to the MeanAMag point. The distance was computed using the centroids from the previous iteration (C_1j−1_, C_2j−1_, C_3j−1_).
  2.6 Centroids are updated using the latest computed sample of the MeanAMag feature characteristic (*Z*_i_).
  2.7 Both the *PME_Classifcation_label* and the *k-means_Classifcation_label* are combined using previously learned decision rules based on collected data from Walton et al. [8]. The process is repeated until no more information is provided. At each new iteration the classification prediction (Y_j_) will be obtained using the centroids from the previous iteration (C_1j−1_, C_2j−1_, C_3j−1_).

In the combined algorithm, k-means generates three different clusters that evolve with an increasing number of samples. Clusters generated by the unsupervised k-means algorithm correspond to three classes (k = 3), namely, high, medium and low MeanAMag, which is the only input feature characteristic. Discrimination between high, medium and low MeanAMag changes with an increasing number of acquired samples. An initial small set of points (100) was used to compute the initial values for the centres of the high, medium and low classes. Afterwards, for every new MeanAMag value acquired, a high, medium, or low MeanAMag class is predicted based on the Euclidean distance to the previously computed centre values. The newly acquired MeanAMag is then used to update the centres of the three different classes (high, medium and low). 

**Figure 5:**
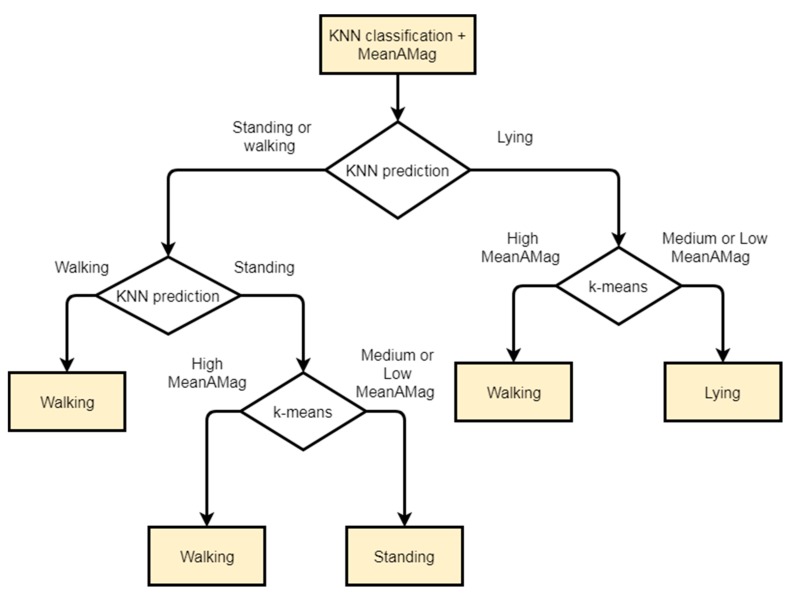
Decision tree rules for the combination of both KNN and k-means algorithms.

Outputs of both the PME classification and k-means unsupervised clustering were combined using a set of previously learned decision rules. These decision rules were obtained using a decision tree classifier based on previously collected data (Walton et al. [8]). The set of decision rules obtained is shown in Figure 5. Based on these decision rules, clusters in the combined algorithm are formed as:

1. Walking cluster contains samples predicted as walking by the KNN algorithm, samples predicted as standing by the KNN algorithm with a high MeanAMag value by the k-means algorithm, and samples predicted as walking by the KNN algorithm with a high MeanAMag value by the k-means.
2. Standing cluster contains samples predicted as lying by the KNN with a low MeanAMag by the k-means algorithm, and samples predicted as standing by the KNN algorithm with medium or low MeanAMag by the k-means algorithm.
3. Lying cluster contains samples predicted lying by KNN with medium or low MeanAMag prediction by the k-means.

Outputs labels obtained by the k-means unsupervised online learning algorithm are based on an online computation of the centres and hence they are not static. In this scenario, a k-means unsupervised learning algorithm provides a component of the combined algorithm that captures changes in the MeanAMag and adapts accordingly. With the incorporation of the k-means and KNN algorithm it is possible to obtain a feature set that is more representative of the classification problem [20,21]. 

## 3. Experimentation Setup and Operation

This section details the experimentation setup, describing the study site, the data acquisition equipment and data processing methodology. 

### 3.1. Study Site and Animals

**Figure 6:**
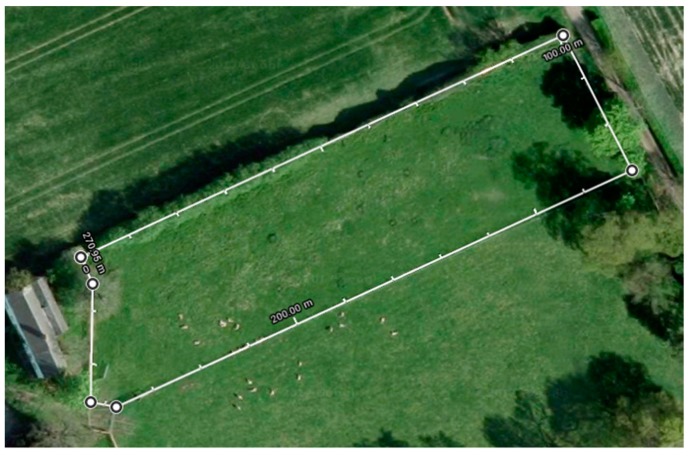
Satellite view of field with drawn and labelled perimeter.

Before starting the main trial, a pilot study was conducted for 3 days to check the research protocols described below and also to test battery life and operation of the Intel® SE C1000 device in the field. Battery life of the 270 mAh battery during the pilot was 2.4 days (±2 h) as no restrictions/strategies were enforced. Ethical permission was obtained for the School of Veterinary Medicine and Science, University of Nottingham (unique reference number 1481 150603). For the main trial in this study, data was collected for 39 days from 19 July 2017 to 4 October 2017. A total of 26 sheep were selected via stratified random sampling (age) from a flock of 140 animals at the University of Nottingham. Assessment of body condition, age and breed was done on the first day of the trial. Body condition scoring of sheep is simply a means of assessing the degree of fatness or condition of the living animal and was scored using UK industry guidelines [33]. The selected sheep had various body condition scores ranging from 2.5 to 4.5 and an age ranging from 1 to 4 years. The breeds of the sheep were Lleyn crosses (13 individuals), Aberfield cross (5 individuals), Exlana crosses (3 individuals), Texel crosses (4 individuals) and a Berichon du Cher (1 individual). Sheep were kept in a rectangular 0.75-acre field with a 270 m perimeter, where observational recordings were taking place (Figure 6). Individual identification was facilitated by spraying a number between 1 and 26 on an animal’s side. Numbers were sprayed regularly (every 2 weeks), to aid identification. From the total number of sheep kept in this field only 17 sheep were used to collect sensor and observation data due to limited human resources available to observe all the animals in the field. Sheep were selected on the basis of including a wide range of body condition, age and breeds.

### 3.2. Data Acquisition Equipment, Physical and Classification Setup

**Figure 7:**
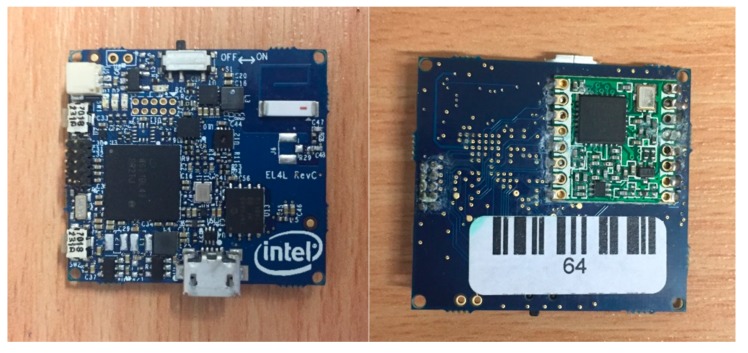
Custom-made device (including processor, memory, radio, inertial measurement unit (IMU), etc.).

Sensor data was collected using a custom-made wearable device (Figure 7), based on the Intel^®^Quark™ SE C1000 microcontroller. In summary, the device encompassed flash memory, a low power wide area (LPWA) radio module, a tri-axial accelerometer and tri-axial gyroscope and a Pattern Matching Engine (PME). Full details of the configuration of the device can be found in Walton et al. [8]. The devices were attached to a light-weight Li-Po battery 270 mAh Li-ion battery. 

The devices were designed to support edge-based processing, classification and radio transmission. A vectorised model based on a K-nearest neighbours (KNN) algorithm (K = 1) [34] was implemented for classification of three relevant behaviours (walking, standing and lying). This model uses a KNN algorithm to label a new sample by comparing its vector to the vectorised model in hardware (the PME) which itself is based on the Walton et al. [8] dataset. 

Walton et al. [8], proposed the use of 11 feature characteristics for each of 4 variables (acceleration magnitude, acceleration magnitude difference, gyroscope magnitude, and gyroscope magnitude difference) making a total of 44 feature characteristics (i.e., 11 features × 4 variables). 

However, in this study, 5 feature characteristics, namely, mean, standard deviation, interquartile range, kurtosis and min, were implemented on the device, i.e., 5 features × 4 variables giving 20 feature characteristics. These features were implemented considering PME design and computation complexity of other features, i.e., frequency domain features are more complex than time domain features, which have a significant impact on power consumption.

Devices were contained in a custom-made 3D printed close fitting plastic enclosure. Once in the enclosure, devices were attached to the sheep’s ear using Velcro and cable ties were used to secure them. All devices were fixed using the same orientation. At the beginning of each trial period, devices were mounted on the sheep, and removed 2 days after, with the cycle repeated. In this mode of operation, recorded data was downloaded over universal serial bus (USB) communication system (as opposed to radio) from the device using software provided by Intel. 

### 3.3. Behaviour Observation Methodology

Behaviour identification was based on an ethogram developed in a previous pilot study [8]. Behaviours of interest for this study were identified according to the following definitions: walking (sheep moves in four beat motion for at least 2 s), standing (sheep is standing on their four legs, head up or down), lying (sheep lying on ground with or without jaw movement). Full details of the ethogram can be found in Walton et al. [8]. Annotations of the observations were recorded into a comma separated values (csv) file with associated timestamps. These annotations were made by a well-trained researcher. Possible discrepancies in the classification performance, due to ground truth annotation mistakes from this study, should be minimal, as walking, standing and lying have an easily observable cut off difference in comparison to other behaviours where this difference is more subtle (e.g., social behaviours). Additionally, behaviours such as lying and standing have very large bout durations and, hence, if there are any misclassified samples they will be at the beginning or the end of the bout behaviour, making their total number very small compared to the total number of samples.

As stated, behavioural information was recorded by two well-trained research technicians. Timestamps of the behavioural annotations were recorded using a stopwatch which was synchronised with the internal clock of a laptop computer and the wearable devices where synched with the same using the low power radio on the devices and a dongle on the computer. Observations were taken in two different sessions: a morning session with 2 h duration (typically between 9:00–11:00 am) and an afternoon session with 1 h duration (typically between 2:00–3:00 pm).

### 3.4. Classification Performance Methodology

Metrics used to evaluate the performance of algorithm classification include accuracy, specificity, recall (also known as sensitivity), precision, and F-score as defined in Dohoo et al. [35].

### 3.5. Data Processing: “In-the-Field Data Gathering”

As outlined in Section 3.2, five feature characteristics, namely, mean, standard deviation, interquartile range, kurtosis and min, were implemented on the device. These feature characteristics were applied to the four variables, namely, acceleration magnitude, acceleration magnitude difference, gyroscope magnitude, and gyroscope magnitude difference, making a total of 20 different feature characteristics.

The devices were mounted at the start of the trial and cycled every 2 days as outlined in Section 3.2. The PME made classifications based on 16 Hz sample data and using a 7 s window size. MeanAMag as described in Section 2.2 was calculated. The resulting dataset contained 3 different variables: (1) timestamp, (2) a PME classification label, i.e., from the offline KNN algorithm/classifier and (3) a devised classification label based on the online MeanAMag calculation. Data containing spurious information or no behavioural observation was removed before analysis. A total of 44 datasets from 17 sheep were utilised for further analysis (dataset 2 in Figure 4).

### 3.6. Data Processing: “Desk-Based Data Preparation”

This in-the-field dataset (dataset 2 in Figure 4) was combined with the observed behaviour classification labels (i.e., ground-truth). The resulting dataset contained 4 different variables: (1) timestamp, (2) PME classification label, (3) MeanAMag label and (4) ground truth behaviour label. 

All these four variables were aligned based on a 10 s window (7 s sampling + 3 s delay for transmission). During the alignment of the two different datasets (sensor data and ground truth observations) an individual class label was assigned to the observation records. Hence, behavioural observations were discretised using a 10 s window. 

In cases where all the samples in the data belong to the same class, the ground truth class was set to that particular behaviour. For windows that contained more than one behaviour label, the majority class was set as the ground truth label. 

### 3.7. Data Processing: “K-Means + Combined Algorithm”

Processing of the data was performed using custom made scripts written in Python 3.5 [36] and Matlab 2017a [30]. The combined approach uses as an input *PME_Classifcation_label* and MeanAMag and generates a classification output prediction as described in Section 2.3. Ground truth observations were used to evaluate the combined algorithm. 

## 4. Results

In order to compare the performance of the combined algorithm against individual offline KNN only and online *K*-means only algorithms, three different evaluations are performed: (1) offline KNN algorithm only, (2) online k-means algorithm only and (3) combined offline and online algorithm.

### 4.1. Performance of the Classification Using only the Offline KNN Algorithm

In this study, the KNN algorithm, which was trained offline with previously collected data from Walton et al. [8]), was utilised within this study. As stated, the C1000 devices are equipped with a PME that enables implementation of the KNN algorithm, thus providing a classification on real time sampling based on the vectorised offline KNN model. As mentioned before, classification was performed using a total of 20 feature characteristics. The classification performance obtained with this approach when tested using data collected from the Walton et al. study [8], is shown in Table 1. 

The performance of the same classifier on data collected for this study is shown in Table 2.

Classification performance using the offline KNN algorithm/classifier (first trial, Table 1) was very poor when applied to data in this trial, with and overall accuracy of 48.08% (Table 2) and is evidence of the very issue of concept drift this study aimed to address. The worst results of the classification were obtained for standing and walking with values on accuracy of 31.07% and 33.15%, respectively. The best performance was obtained for lying with an accuracy of 80%, and specificity of 94.56%. However, values were low for recall (44.04%) and F-score (55.92%) for this activity. This represented an average decrease of 35.41% in accuracy, 20.19% in specificity, 26.19% in recall, 37.39% in precision, and 47.45% in F-score with the initial expected performance (Table 1).

### 4.2. Performance of the Classification Using only Online K-Means Algorithm

The performance of the online K-means algorithm evaluated against data collected for this study, are shown in Table 3. Performance was evaluated on the basis of accurate predictions by data sample. 

Performance of the classification using only the online learning algorithm was better than the offline KNN algorithm (as applied in this study), with an overall 69.49% accuracy. The lowest values were obtained for standing (56.49% in accuracy, and 40.29% in recall), for walking (17.58% in recall, 54.46% in precision, and 26.58% in F-score). 

An average decrease of 14% in accuracy, 11.33% in specificity, 23.35% in recall, 15.18% in precision, and 28.16% in F-score was seen when comparing the performance of the offline KNN algorithm (Table 1) as applied in the original Walton et al study and the performance using only the online *k*-means algorithm as applied to the data in this study.

### 4.3. Performance of the Combined Algorithm 

The performance of the combined algorithm was evaluated using ground truth observations and predictions from the algorithm, with each of them being at 10 s windows. Performance was evaluated on the basis of accurate predictions by data sample. Results of the classifier performance of the combined algorithm are shown in Table 4. 

When evaluating the combined algorithmic approach, the best accuracy was produced for walking (92.93%), and the worst accuracy for standing (78.25%). Similarly, the highest specificity values were obtained for walking (98.87%) and the worst for standing (58.16%). However, the worst recall (17.22%), precision (54.56%) and F-score (26.18%) values were obtained for walking. An average increase of 1.69% in accuracy, a decrease of 4.71% in specificity, a decrease of 16.25% in recall, a decrease 4.63% in precision and 13.71% decrease in F-score were obtained when comparing performance to the original pre-trained KNN algorithm (Table 1). Nevertheless, the combined method offers an improvement when comparing with the offline KNN method with an average increase of 37.10% in accuracy, 15.48% in specificity, 9.94% in recall, 33.26% in precision and 33.74% in F-score. Similarly, when compared to the online *k*-means algorithm, the combined algorithm provides an increase of 15.69% in accuracy, 6.62% in specificity, 7.10% in recall, 10.55% in recall and 14.45%. These results show that the combined algorithm is better that both the offline implemented algorithm and the online *k*-means algorithm.

### 4.4. Distribution Change in MeanAMag

**Figure 8:**
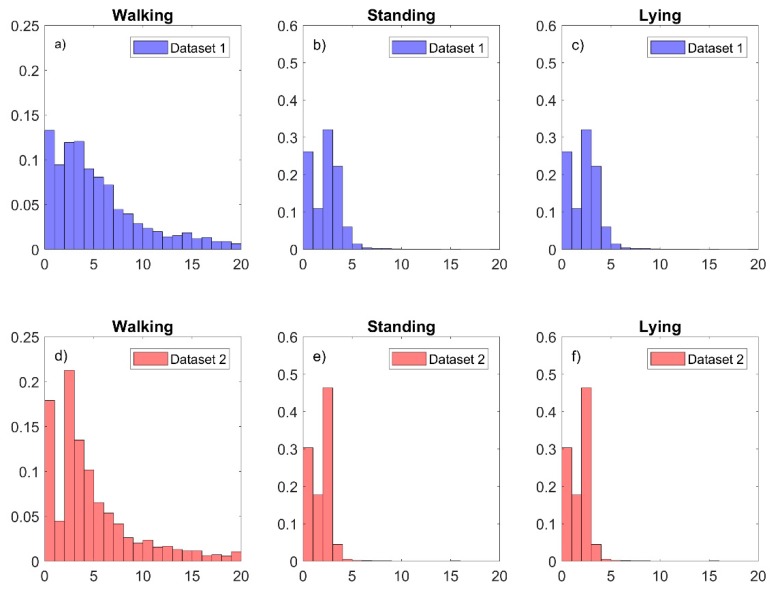
Distribution of the MeanAMag for our previously collected dataset [8] (dataset 1) and for this study (dataset 2). The plot shows the distribution change between the two studies for the three different behaviours. (**a**–**c**) show the distributions of the MeanAMag for walking, standing and lying using collected dataset [8], (**d**–**f**) show the distribution of the MeanAMag for this study.

Distribution change in MeanAMag between the initially collected dataset [8] and data collected for this study is shown in Figure 8.

Figure 8 shows a drift in the distribution for the MeanAMag occurred between previously collected data [8] and data collected in this study. In general, there was a decrease in MeanAMag for the current study. More specifically, for walking sheep behaviour, the MeanAMag decreased from an average 5.74 (SD 6.39) in dataset 1 to a 4.23 (SD 4.20) in the current study. For standing sheep behaviour, MeanAMag decreased from an average 1.83 (SD 1.57) in dataset 1 to 1.31 (SD 1.17) in dataset 2. For lying sheep behaviour, MeanAMag decreased from an average 0.60 (SD 1.45) in dataset 1 to a 0.20 (SD 0.80).

### 4.5. Evolution in Cluster Centres

**Figure 9:**
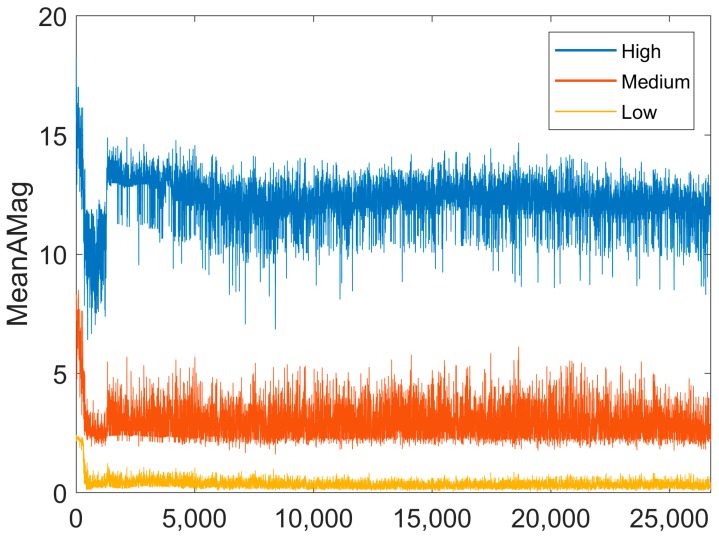
Evolution of the centres of the different classes computed using the k-means online learning algorithm with data collected for this study. The k-means online learning algorithm will discriminate between high, medium and low MeanAMag values.

Figure 9 shows the evolution of the centre for each of the behaviours obtained by the k-means online learning algorithm. In general, there was a rapid decrease from sample points 1 to 300 and then a rapid increase from 1200 to 1300, after which all centres plateau. Centres computed from the previously collected data [8] were 16.66, 3.28 and 0.81 for walking, standing and lying, respectively. 

## 5. Discussion

Although there are many algorithmic approaches developed for precision livestock farming, very rarely have these been tested in the field. Discrepancies in classifier performance when evaluating in different environments have been reported very frequently in human studies [17,18]. Additionally, recent livestock research has pointed to the lack of feature correspondence across trials/sensors, which leads to poor usability or applicability (i.e., the inability to identify corresponding features across studies/modalities means we have no generally applicable means of classification) [20,21]. To the author’s knowledge, this is the first study in precision livestock that provides an offline and online algorithmic approach that successfully deals with concept drift.

One of the biggest challenges for long-term behavioural monitoring systems is how to develop flexible systems that can adapt to different sets of conditions (i.e., different terrain) which might have a significant effect on the performance of the monitoring system. For example, in HAR, discrepancies in classifier performance have been found when validating a system in an unrestricted environment with a classifier trained in a restricted environment [17]. Similarly, large decrements have been reported when comparing the performance of a physical activity classifier in humans between laboratory and real-life scenarios [16]. Monitoring systems that do not incorporate such discrepancies can lead to infer behaviour with a large percentage of error. In this study, we found a large decrement from the expected classification performance of sheep’s behaviour (35.41% in accuracy, 20.19% in specificity, 26.19% in recall, 37.39% in precision, and 47.45% in F-score) to the performance of the same algorithm in a different environmental set up. Such discrepancies in the performance of the classification might be due to differences in the animals, and the environment from which the data was collected from (e.g., breed, age animals, terrain of farm, soil composition, etc.). For example, the on-chip implemented algorithm was based on data from 6 different sheep with Texel cross, Suffolk cross and Mule breed, whereas the current study also includes Lleyn, Aberfield cross, Exlana, Berichon du Cher. Additionally, in the previous study [8], age was between 18 months to 4 years, whereas the current study includes sheep with an age ranging from 1 to 4 years. Moreover, there were field differences (with respect to size and type) in the previous study [8] and the current study. 

Systems that considered evolving data streams in HAR have been recently developed [13,14,15]. However, to the author’s knowledge, animal behaviour classification systems that incorporate possible changes in the distribution of classes have not yet been developed. The combined algorithm presented in this study incorporates an offline classifier and an online algorithm providing the flexibility to deal with such possible distribution changes via the online algorithm, while preserving the already learned knowledge with the offline algorithm. Similar to the results obtained in this study, combined offline classifier and online algorithms have shown a performance improvement when compared to classification using only offline or only online algorithms, for example: visualisation [22], recognition of handwritten digits [37] and classification of electrocorticography [38]. The performance of the combined offline classifier and online algorithm was slightly reduced relative to the pre-trained KNN algorithm, with a 7.52% lower performance (average across the different performance metrics). However, the combined algorithm shows a 25.90% higher performance when compared to using the offline algorithm only, and a 10.88% higher performance when using the online algorithm only. Neither the offline algorithm alone nor the online algorithm alone replicated the expected performance of the KNN pre-trained algorithm. Moreover, average accuracy (85.18%) of the combined algorithm was better than the average accuracy for the pre-trained KNN algorithm. In particular, accuracy was higher for walking (92.93%) using the combined algorithm than in the pre-trained KNN (82%).

One limitation of the combined algorithm is the relatively poor recall (57.82% on average) and precision (69.9% on average) which are mainly due to poor recall (17.22%) and precision (54.56%) in walking. In this respect, depending on the application or use-case of the technology a minimum classification performance might be required. For example, for detecting lameness within walking in might be necessary to first obtain a higher level of recall (sensitivity) and precision for this behaviour. However, one major highlight of the results of the combined algorithm is the fact that using only one feature characteristic the (MeanAMag) as an input to the online learning algorithm can yield such an improvement relative to the pre-trained KNN algorithm. Additionally, by incorporating an online unsupervised clustering algorithm (online k-means) the combined algorithm is able to capture possible changes in the distribution of the different classes and hence it will be more flexible with challenging new or changing conditions, and might show higher performance than statistical offline methods. Such changes in distribution (“concept drift”) can lead to differences in the performance of a classifier, such as that recently reported by Guo et al. [19] with grazing behaviour of sheep on pastures with different sward heights, and the discrepancies on the performance of classification of behaviour using pedometers reported by Ungar et al. [18]. Moreover, by incorporating other feature characteristics as an input to the online learning algorithm it might be possible to further improve the performance of the combined offline and online algorithms. In this study, only the MeanAMag feature characteristic was retrieved in order to minimise the energy consumption used in writing each individual variable to flash, as previously reported in Walton et al. [8]. Once again, even when the inclusion of more feature characteristics can have a positive impact, additional feature characteristics should be incorporated on a cost-benefit basis, since each individual feature characteristic increases the computational cost and different features have a different computational cost. 

Due to its embedded architecture and computational power, it should be feasible to implement the combined offline and online learning algorithm on the SE C1000 for future evaluation in different changing conditions (i.e., different farms, different breeds, etc.). Such an implementation should consider the computational cost of the *k*-means learner used for the online learning in this study, against other online learning algorithms that might have lower computational cost. Whilst in this study we have designed a combined offline and online learning that classifies with a high performance level (overall average accuracy of 85.18%) for biologically relevant activities (walking, standing and lying) in sheep, in our future work, we will need to implement such a combined algorithm on the device and further validate the system under different changing conditions. Additionally, future work can incorporate the addition of gyroscope features for the online learning algorithm as well as more accelerometer features. 

## 6. Conclusions

The results from this study show that by using a combined offline trained classifier and online learning algorithmic classifier approach, it is possible to accurately classify (78.35% to 92.93%) relevant behaviours (walking, standing and lying) in sheep when presented with new and changing conditions. Evaluation of the combined algorithm in this use-case resulted in average accuracies of 85.18%, average specificities of 82.84%, average recall of 57.82%, average precision of 69.9% and average F-score of 60.4%. The combined algorithm outperformed both the offline classifier (average increase of 25.90%) and online algorithm (average increase of 10.88%). The combination of offline classifier and online algorithm employs a set of rules based on previously collected data. This method provides the necessary information for the system to adapt to possible distribution changes of the class behaviours. Due to its flexibility, the combined algorithm can be used to accurately classify walking, standing and lying in changed conditions (breed, age, body condition and in different environments) in sheep. Therefore, the system (device and algorithm) represents a potential solution for real-time and long-term automated monitoring within a precision livestock approach. 

## Figures and Tables

**Table 1:** Performance of offline KNN algorithm implementation when tested using previously collected data [8]. In italics is the lowest value and in bold is the highest value across the different behaviours.

Performance	Accuracy	Specificity	Recall	Precision	F-score
Walking	82.00	**90.32**	*60.95*	71.37	*65.75*
Standing	*79.07*	*83.14*	71.43	*69.33*	70.37
Lying	**89.42**	89.18	**89.83**	**82.90**	**86.22**
Average	83.49	87.55	74.07	74.53	74.11

**Table 2:** Performance of the classification using only prediction from the implemented KNN offline algorithm. In italics are the lowest values for each measure and in bold is the highest across the different behaviours.

Performance	Accuracy	Specificity	Recall	Precision	F-score
Walking	33.15	*28.09*	96	*9.79*	17.63
Standing	*31.07*	79.42	*3.62*	23.76	*6.28*
Lying	**80**	**94.56**	**44.04**	**76.57**	**55.92**
Overall	48.08	67.36	47.88	36.64	26.66

**Table 3:** Performance of the classification using only the online learning algorithm. In italics is the lowest value and in bold is the highest value across the different behaviours.

Performance	Accuracy	Specificity	Recall	Precision	F-score
Walking	**92.93**	**98.85**	***17.58***	54.46	*26.58*
Standing	*56.49*	85.07	40.29	**82.64**	54.17
Lying	59.06	*44.74*	**94.28**	*40.95*	**57.10**
Overall	69.49	76.22	50.72	59.35	45.95

**Table 4:** Performance of the combined offline and online algorithm. In italics is the lowest value and in bold is the highest value across the different behaviours.

Performance	Accuracy	Specificity	Recall	Precision	F-score
Walking	**92.93**	**98.87**	*17.22*	***54.56***	*26.18*
Standing	***78.35***	***58.16***	**89.79**	**79.11**	**84.11**
Lying	84.25	91.48	66.45	**76.03**	70.92
Overall	85.18	82.84	57.82	69.90	60.40
